# Interleukin-36 is vasculoprotective in both sexes despite sex-specific changes in the coronary microcirculation response to IR injury

**DOI:** 10.3389/fcvm.2023.1227499

**Published:** 2023-09-11

**Authors:** Juma El-Awaisi, Joanne L. Mitchell, Aaron Ranasinghe, Neena Kalia

**Affiliations:** ^1^ Microcirculation Research Group, Institute of Cardiovascular Sciences, College of Medical and Dental Sciences, University of Birmingham, Birmingham, United Kingdom; ^2^ Consultant Cardiac and Heart/Lung Transplant Consultant, Queen Elizabeth Hospital, University Hospitals Birmingham NHS Trust, Birmingham, United Kingdom

**Keywords:** myocardial infarction, coronary microcirculation, sex, ischaemia-reperfusion injury, neutrophils, platelets, interleukin-36

## Abstract

**Aims:**

Risks and outcomes of myocardial infarction (MI) are different between men and women and some studies have demonstrated that the latter have a higher risk of mortality. Whilst there are many reasons for this, it may also partially be linked to stronger innate and adaptive immune responses mounted by females compared to males. However, little is known about how sex impacts the coronary microvessels, the site where inflammatory processes take place, after an MI. Intravital and laser speckle microscopy was used to image coronary microvessels and ventricular perfusion *in vivo* in response to myocardial ischaemia-reperfusion (IR) injury in male and female mice. Interleukin-36 (IL-36) is the latest addition to the IL-1 superfamily of pro-inflammatory cytokines and has recently been shown to mediate inflammation in a number of non-cardiovascular diseases. Its role in mediating potential sex-related microcirculatiory pertubations in the heart are unknown. Therefore, the vasculoprotective efficacy of an IL-36 receptor antagonist (IL-36Ra) was also investigated.

**Methods and results:**

Immunostaining and flow cytometry demonstrated higher expression of IL-36 and its receptor in female hearts, an observation confirmed in human samples. Intravital imaging of the anaesthetised mouse beating heart identified significantly greater neutrophil recruitment in female hearts, but a greater burden of thrombotic disease in male hearts. Male mice had reduced functional capillary density and were unable to restore perfusion to baseline values as effectively as females. However, female mice had significantly larger infarcts. Interestingly, IL-36Ra decreased inflammation, improved perfusion, and reduced infarct size in both sexes despite increasing platelet presence in male hearts. Mechanistically, this was explained by IL-36Ra attenuating endothelial oxidative damage and VCAM-1 expression. Importantly, IL-36Ra administration during ischaemia was critical for vasculoprotection to be realised.

**Conclusion:**

This novel study identified notable sex-related differences in the coronary microcirculatory response to myocardial IR injury which may explain why some studies have noted poorer outcomes in women after MI. Whilst contemporary MI treatment focuses on anti-platelet strategies, the heightened presence of neutrophils in female IR injured coronary microvessels necessitates the development of an effective anti-inflammatory approach for treating female patients. We also emphasise the importance of early intervention during the ischaemic period in order to maximise therapeutic effectiveness.

## Graphical Abstract

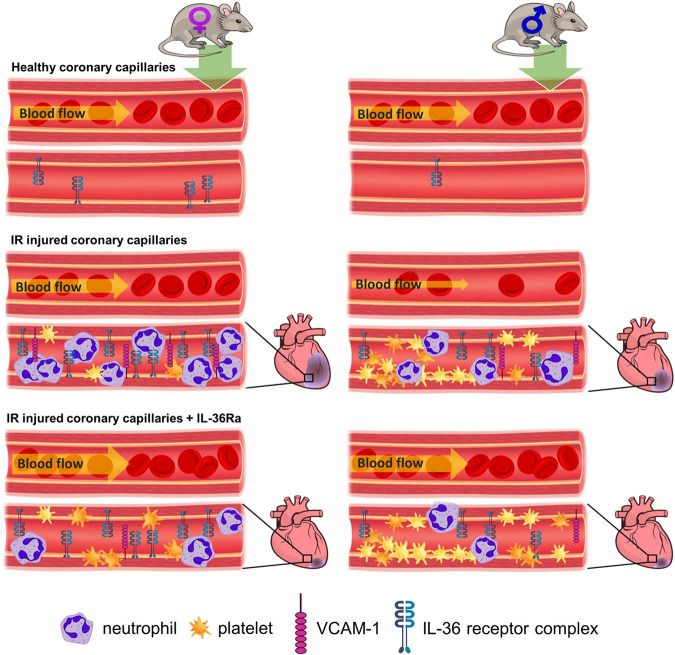

## Introduction

The primary objective in treating ST-elevation myocardial infarction (MI) is to swiftly restore blood flow after the occurrence of a blockage in one or more of the epicardial coronary arteries. This is accomplished through primary percutaneous coronary intervention (PCI) procedures that utilize a stent to open the affected artery. However, despite these treatments, a significant number of patients suffer extensive muscle damage and subsequently develop heart failure (1). This can be attributed, in part, to reperfusion causing additional tissue damage, a phenomenon called ischaemia-reperfusion (IR) injury. Indeed, sub-optimal myocardial perfusion is observed in up to 50% of patients after successful PCI, leading to poorer outcomes compared to those with complete perfusion (2). This suggests that tissue injury likely occurs as a result of insufficient coronary microcirculatory perfusion (3, 4). Unfortunately, current clinical imaging methods are incapable of visualizing microvessels smaller than 200 μm, leaving much unknown about the extent of coronary microcirculatory responses to IR injury.

Some studies have suggested that biological sex can potentially determine patient outcomes following cardiovascular diseases (CVDs) independent of “traditional” risk factors. For example, men have twice the risk of suffering an MI and present at a younger age than women (5). This has often been misinterpreted as females being “protected” against heart disease. However, women have a longer stay in hospital and have a higher incidence of in-hospital, 30-day and 1-year mortality post-MI (6, 7). There are a number of reasons that can explain these differences including the fact that women have a higher risk profile and greater comorbidity burden (e.g., diabetes, hypertriglyceridemia, metabolic syndrome) than men on presentation and are also older at the time of presentation (8). However, it is also possible that stronger innate and adaptive immune responses mounted by females compared to males may also contribute to different cardiovascular outcomes (9). Despite numerous studies highlighting marked differences between males and females in their immune function, sex-related differences in the pathophysiology of CVDs has historically been overlooked in experimental investigations. Hence, little is known about how biological sex impacts thromboinflammatory responses, specifically within the coronary microcirculation *in vivo*, and whether it increases the likelihood of microvascular disturbances post-reperfusion injury.

Interleukin-1 family members are major mediators of sterile inflammation, acting at the apex of inflammatory cascades that ultimately trigger the production of a plethora of cytokines/chemokines from diverse cell types. Therefore, these factors may significantly contribute to microcirculatory disturbances in the myocardium after a heart attack (10). Indeed, encouraging results were observed in the large-scale canakinumab anti-inflammatory thrombosis outcomes study (CANTOS) trial, in which blocking IL-1 improved long-term outcomes following MI (11). Interleukin-36 (IL-36) is a new addition to the IL-1 family sharing structural and functional similarities with IL-1 (12, 13). IL-36, consisting of three agonist ligands (IL-36α, IL-36β, and IL-36γ), is emerging as a novel regulator of both innate and adaptive immune responses in various acute and chronic conditions. Alongside enhancing the effects of IL-1, IL-36 itself is a potent inflammatory mediator (14).

We were the first to demonstrate a new and critical pathological role for the IL-36/IL-36R signalling pathway in mediating coronary microcirculatory perturbations in adult female mice after myocardial IR injury. Indeed, targeting the IL-36 receptor (IL-36R) with a receptor antagonist (IL-36Ra) was a highly effective and novel vasculo- and cardioprotective intervention (15). Importantly, we showed that this therapy remained effective in the aged female mouse heart where heightened coronary perturbations were noted after IR injury (15). However, experimental testing of novel therapeutic candidates in both sexes has historically been neglected. This is particularly important for therapies targetting inflammatory cytokines as sex-related differences in cytokine secretion and expression are well known. However, it is currently not known whether there are sex-related differences in the myocardial expression of IL-36/IL-36R, in the responsiveness of male and females to IL-36Ra treatment and whether targeting this pathway can be therapeutic against myocardial IR injury in both sexes.

We previously described an intravital approach that allowed the coronary microcirculation of the mouse beating heart to be imaged *in vivo* with cellular resolution from 15 min post-reperfusion. In this study, we optimised our imaging technique to enable imaging immediately post-reperfusion and, combined with laser speckle contrast imaging (LSCI), used it to investigate whether the observed poorer outcomes in women following post-MI were associated with a heightened susceptibility of female coronary microvessels to reperfusion injury. We also examined the expression of IL-36 and IL-36R in heart tissue from mice and humans, as well as in mouse neutrophils of both sexes. Additionally, we gained mechanistic insights into how IL36Ra may provide vascular protection by reducing ROS-induced oxidative damage, and by inhibiting the expression of vascular cell adhesion molecule-1 (VCAM-1).

## Methods

### Myocardial IR injury and beating heart intravital imaging

Adult male and female C57BL/6 mice (2–4 months) were used in accordance with the Animals (Scientific Procedures) Act of 1986 (Project licence P552D4447). To induce anesthesia, ketamine hydrochloride (100 mg/kg) and medetomidine hydrochloride (100 mg/kg) were administered intraperitoneally. Myocardial IR injury and was performed as previously described (15). Briefly, the left anterior descending (LAD) artery was tied for 45 min, followed by reperfusion for specific time intervals: 2 h for tissue analysis, 2.5 h for intravital observations, 4 h for infarct measurement, and different time points (0, 30, 120, or 150 min) for flow cytometry analysis. It was possible to keep imaging the beating heart for at least 3 h before complications sometimes arose due to mice being under injectable anaesthesia long term. Therefore, images were captured for 2.5 h post-reperfusion to maximise data collection from each mouse. The duration of reperfusion had to be increased to 4 h for infarct quantification to allow clear delineation of infarcts.

The time of intervention is a crucial factor affecting the clinical efficacy of anti-inflammatory drugs. Microvascular no-reflow develops rapidly following reperfusion, making the initial hours or even minutes critical. To address this, our treatment strategy involved administering the antagonist (recombinant mouse IL-36Ra—15 ug/mouse; Novus Biologicals) either during the ischaemic phase (35 min of ischaemia), the reperfusion phase (60 min after reperfusion), or at both time points.

Real-time intravital observations were performed as previously described (15). Briefly, a 3D printed stabilizer was fixed downstream of the ligation site on the left ventricle. PE anti-mouse Ly-6G (Biolegend) and APC anti-mouse CD41 (Biolegend) antibodies were injected 10 min before reperfusion to visualize neutrophils and platelets. FITC-BSA (Sigma) was injected after a 2.5 h reperfusion period to assess vascular perfusion and FCD in separate mice. Intravital imaging allowed microvascular events on the heart's surface to be captured at a depth of approximately 50–60 µm.

### Multiphoton imaging of heart sections

To determine whether intravital observations captured from the heart surface were mirrored throughout the ventricular wall, multiphoton microscopy was performed on harvested hearts. The left ventricle was vibratome (Campden Instruments Limited, UK) cut into four 300 µm sections and multiphoton (FVMPE-RS Olympus) images were obtained from the epicardial to the endocardial end. Z-stacks from each section were processed into 3D stack images and the presence of neutrophils was analysed as the sum fluorescence intensity for each section (Using Image J).

### Immunohistochemistry analysis of IL-36 cytokines and IL-36R in mouse tissue

Frozen heart tissue sections (10 µm) were incubated with primary antibodies against IL-36R, IL-36α, IL-36β, or IgG control antibodies (1:100 dilution; R & D Systems) and a secondary donkey anti-goat Alexa Fluor-488 antibody (1:100 dilution; Abcam) at room temperature. Sections were also incubated with PE anti-mouse CD31 antibody (1:100 dilution, Biolegend), Alexa Fluor-647 anti-mouse CD106/VCAM-1 antibody (1:100 dilution, Biolegend), or an anti-DNA/RNA damage antibody for oxidative damage detection (1:100 dilution, Abcam). Images were captured using an EVOS FL microscope (ThermoFisher Scientific), and ImageJ was used to quantify mean fluorescence intensity (MFI) for each image.

### Immunohistochemistry analysis of IL-36 cytokines and IL-36R in human tissue

Left ventricular tissue was collected from male and female patients (48 ± 12 years old; Table 1) undergoing implantation of a left ventricular assist device (LVAD) for heart failure. Ethical approval was obtained by the Human Biomaterial Resource Centre at the University of Birmingham (15/NW/0079) and approved by the internal Access Review Panel (19–352). Briefly, a section from the LV apex, removed to allow LVAD implantation, was washed in cold saline and promptly snap-frozen in liquid nitrogen. 10 µm sections of frozen tissue were then stained as described above with the appropriate human antibodies (IL-36R, IL-36α/β/γ, CD31, cTnT, and DNA/RNA damage antibody).

**Table 1:** Demographics of the patients whose left ventricle tissue samples were immunostained for IL-36R and IL-36α/β cytokines. Samples were collected between February 2021 and December 2021 from patients undergoing left ventricular assist device implantation for heart failure. Ethical approval was obtained from the Human Biomaterial Resource Centre at the University of Birmingham (15/NW/0079) and approved by an internal Access Review Panel (19–352).

	Male			Female		
Age	29	50	56	42	54	65
Height (cm)	177	185	178	159	158	185
Weight (kg)	105	103.85	101.15	87.4	76.9	83.45
Ischaemia Time (mins)	78	92	90	102	90	85

### Flow cytometric analysis of endothelial and cardiomyocyte oxidative stress

Flow cytometric analysis of endothelial and cardiomyocyte oxidative stress and IL-36R was performed as previously described (15). Briefly, hearts were collagenase-digested to obtain single-cell suspensions. Cells were incubated with antibodies: anti-DNA/RNA damage (1:100 dilution, Abcam), anti-IL-36R (1:100 dilution, R & D Systems; Alexa-647 secondary, 1:100 dilution, Biolegend), anti-CD31 (1:100 dilution, Biolegend), anti-cTnT (1:100 dilution, Miltenyi Biotec), and Zombie dye (1:500 dilution, Biolegend), along with appropriate IgG controls. Flow cytometry using a CyAn™ ADP instrument (Beckman Coulter, USA) captured 250,000 events per sample. Data analysis was performed with Summit 4.3 software (Beckman Coulter, USA).

### Flow cytometric analysis of murine bone marrow (BM)-derived leukocytes

Mouse leukocytes were isolated from the BM as previously described (16). Briefly, legs were excised from mice after either sham, ischaemia only or IR injury surgeries. BM was collected from femurs and tibias into expansion media (DMEM, high glucose supplemented with 8.9% FBS, 1% MEM-D-valine, 0.1% sodium bicarbonate, 1% penicillin/streptomycin, 1% MEM non-essential amino acids, 1% MEM vitamin mix, 10 U/ml interferon-γ pH 7.4). Erythrocytes were lysed using ACK, the suspension washed twice with expansion media and resuspended into PBS containing 0.1% BSA. Cells were then incubated for 1 h at 4°C with primary antibodies towards IL-36R, IL-36α/β and appropriate IgG controls. Cells were washed with PBS containing 0.1% BSA before incubating with AF647-labelled donkey anti-goat. Leukocyte sub-types were identified using fluorescently labelled antibodies towards neutrophils (PE anti-mouse Ly-6G/GR-1; Biolegend) and monocyte/macrophages (PE-Cy7 Ly6C; Biolegend). Antibodies identifying leukocyte populations were split into separate samples to avoid spectral overlap and then fixed with 2% formyl saline. 50,000 events were collected on a Beckmann Coulter CyanADP with compensation performed on the Summit 4.4.00 software. Data was analysed using FlowJo v10.7.2.

### Laser speckle contrast imaging of the beating mouse heart

LSCI quantitated left ventricular myocardial blood flow as previously described (17). Mice were surgically prepared as described earlier and the LSCI device (moorFLPI-2; Moor Instruments, UK) was positioned above the exposed heart. A demarked area, downstream of the LAD ligation site, was identified for collection of flux data during pre-ischaemia, ischaemia, and post-reperfusion. A total number of 1,000 frames were captured at each time point using the manufacturer supplied image software (mFLPI2Measure V2.0; mFLPIReview V5.0) at a frame rate of 25 Hz and using spatial processing (sliding window, time constant: 0.1 s). Basic Speckle Analysis software (SpAn), written in-house allowed identification and collation of flux values during diastole for each time point.

### Myocardial infarct size analysis

After 4 h of reperfusion, the LAD artery was re-ligated, and 0.5% Evans blue dye (Sigma) marked the area at risk (AAR). The heart was then sliced, treated with TTC (Sigma), and imaged using a stereomicroscope. ImageJ analysis quantified the infarct size (TTC-negative white regions) as a percentage of the AAR (TTC-positive red regions/Evans blue-negative).

### Statistical analysis

Statistical analysis was performed using GraphPad 7.0 software. Data was firstly tested for normality prior to using a Student's *t*-test. A one-tailed Student's *t*-test was used to directly compared two groups and one-way or two-way ANOVA with Tukey's post-hoc was used to analyse multiple groups. Area under the curve (AUC) was calculated for time-course experiments and used for subsequent analysis as a summation of the entire period. Data are presented as mean ± SEM, with *p* < 0.05 indicating statistical significance.

## Results

### Cardiac IL-36R and IL-36α/β increases in both sexes with IR injury but is higher in females

**Figure 1:**
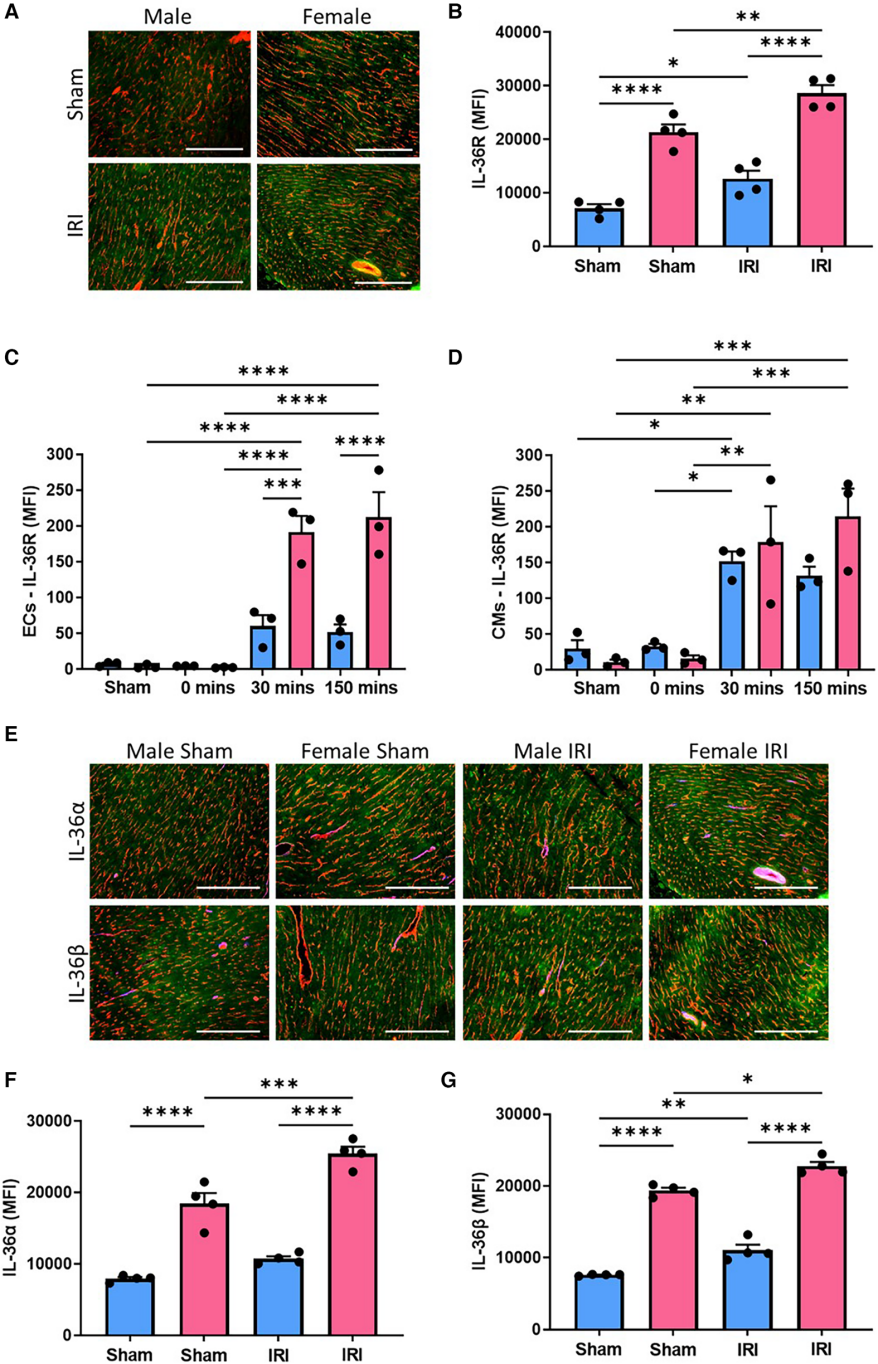
Cardiac expression of IL-36R and IL-36α/β increased with IR injury in both sexes but was generally higher in female mice. (**A**) Representative images of frozen heart sections from male and female sham and injured mice immunostained with an anti-IL-36R (green) and anti-CD31 (red) antibody. (**B**) Quantitative analysis of these images for IL-36R expression. *N* = 4/group. (**C,D**) Hearts were collected from male and female sham mice, after ischaemia only (0 min) and after reperfusion (30 min and 150 min), collagenase digested and analysed flow cytometrically for IL-36R expression on endothelial cells and cardiomyocytes. *N* = 3/group. (**E**) Representative images of frozen hearts sections from male and female sham and injured mice immunostained with an anti-IL-36α/β (green) and anti-CD31 (red) antibody. (**F,G**) Quantitative analysis of these images for IL-36α and IL-36β expression. *N* = 4/group. Scale bar = 100 µm. *p < 0.05, **p < 0.01, ***p < 0.001, ****p < 0.0001 as determined using a two-way ANOVA followed by a Tukey's post-hoc test. All graphs: male = blue; female = pink.

Immunofluorescence staining demonstrated IL-36R was present in mouse hearts, co-localised with coronary vessels and also present on CMs. Basal expression was minimal but significantly (*p* < 0.0001) higher in female sham hearts when compared to male sham hearts. Although expression was increased further after injury in both male (*p* < 0.05) and female (*p* < 0.01) hearts, expression in female injured hearts was significantly (*p* < 0.0001) higher than in male injured hearts (Figures 1A,B; *N* = 4/group). Flow cytometric analysis of digested mouse hearts also demonstrated minimal IL-36R expression in male and female sham heart ECs and also after ischaemia alone. However, this increased post-reperfusion in both sexes with significantly greater expression noted in female coronary ECs at both 30 (*p* < 0.001) and 150 (*p* < 0.0001) minutes when compared to male coronary ECs (Figure 1C; *N* = 3/group). A similar pattern of IL-36R expression was noted flow cytometrically on CMs (Figure 1D). Immunofluorescence staining for IL-36α/β cytokines revealed a similar pattern of expression to IL-36R with significantly (*p* < 0.0001) greater expression in female hearts both basally and after injury (Figures 1E–G; *N* = 4/group).

### Cardiac IL-36R and IL-36α/β/γ in human hearts is higher in females

**Figure 2:**
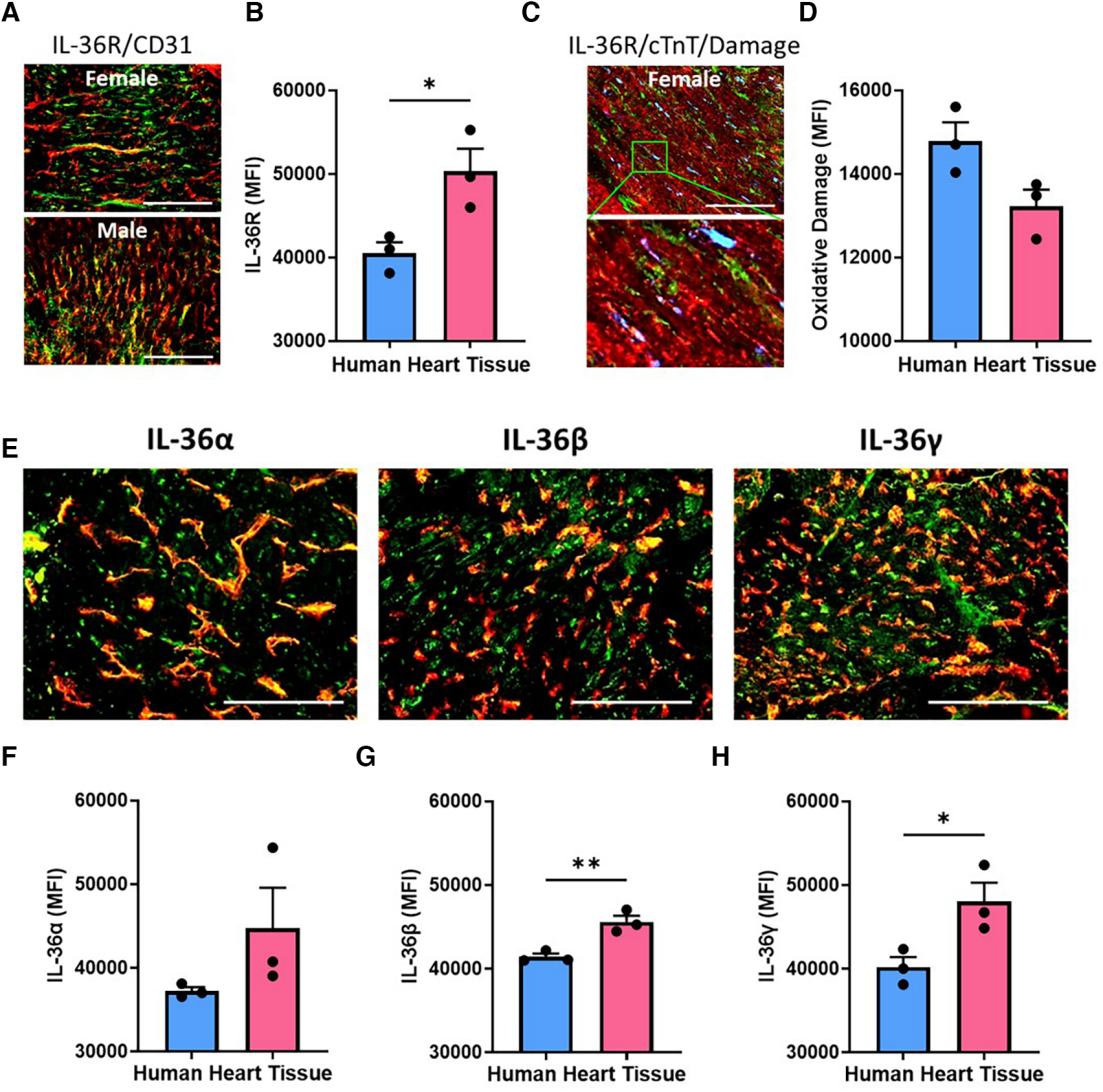
Cardiac expression of IL-36R and IL-36α/β/γ in human heart samples was higher in females. (**A**) Representative images of heart sections from male and female patients undergoing left ventricular assist device (LVAD) implantation and immunostained with an anti-IL-36R (green) and anti-CD31 (red) antibody. (**B**) Quantitative analysis of these images for IL-36R expression. *N* = 3/group (**C**) representative images of a heart section from a female patient undergoing LVAD implantation and immunostained with anti-IL-36R (green), anti-cTnT (red) and anti-DNA/RNA oxidative damage (blue) antibody. (**D**) Quantitative analysis of these images for oxidative damage. *N* = 3/group (**E**) representative images of heart sections from a female patient undergoing LVAD implantation and immunostained with an anti-IL-36α/β/γ (green) and anti-CD31 (red) antibody. (**F–H**) Quantitative analysis of these images for IL-36α, IL-36β and IL-36γ expression. Scale bar = 100 µm. *N* = 3/group. *p < 0.05, **p < 0.01 as determined using an unpaired *t*-test. All graphs: male = blue; female = pink.

Immunofluorescence staining demonstrated IL-36R was also expressed in human hearts, again co-localised with coronary vessels. Expression was significantly (*p* < 0.05) higher in female hearts when compared to male hearts (Figures 2A,B; *N* = 3/group). IL-36R was also detected on CMs and occasionally co-localised with regions in which oxidative damage was present (Figures 2C,D). IL-36α, IL-36β (*p* < 0.01) and IL-36γ (*p* < 0.05) cytokine expression was also higher in female heart sections when compared to male hearts (Figures 2E–H; *N* = 3/group).

### Neutrophil IL-36R and IL-36α/β increases in both sexes with IR injury but is higher in males

**Figure 3:**
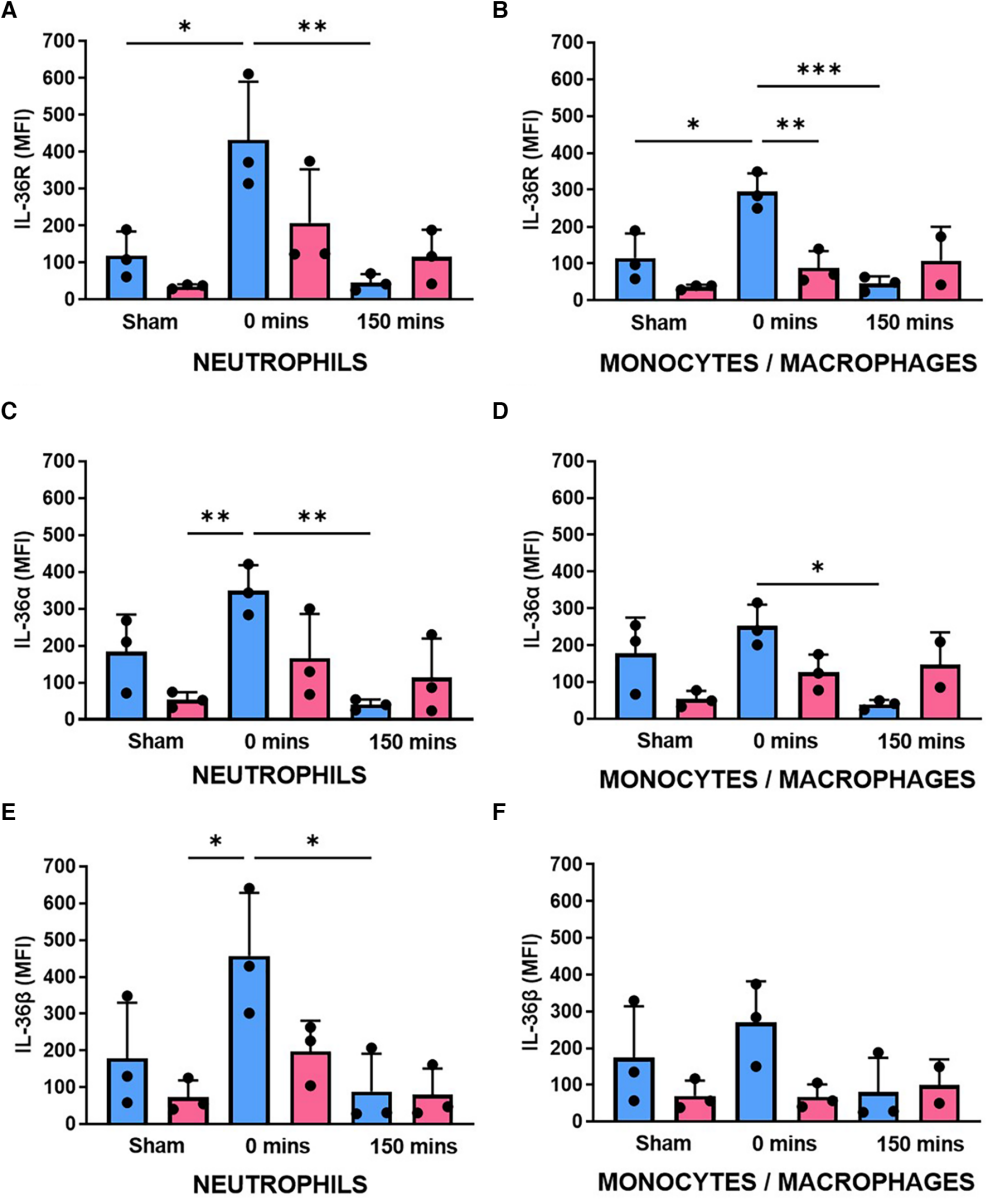
Neutrophil expression of IL-36R and IL-36α/β increased with IR injury in both sexes but was generally higher in male mice. BM-derived neutrophils and monocytes/macrophages were isolated from male and female sham mice, after ischaemia only (0 min) or after reperfusion (150 min), collagenase digested and analysed flow cytometrically for (**A,B**) IL-36R, (**C,D**) IL-36α and (**E,F**) IL-36β expression. *N* = 3/group. *p < 0.05, **p < 0.01, ***p < 0.001 as determined using a one-way ANOVA followed by a Tukey's post-hoc test. All graphs: male = blue; female = pink.

Flow cytometry revealed that IL-36R and IL-36α/β cytokines were also expressed on the surface of mouse BM-derived neutrophils. No significant differences in basal expression was noted between the sexes although there was a trend for higher expression in male neutrophils. Ischaemia increased their expression in both sexes although this was to a greater degree and statistically significant in male mice. In contrast, there was a significant reduction in receptor and cytokine expression after 150 min of reperfusion in both sexes (Figures 3A,C,E; *N* = 3/group). A similar pattern of events, but less marked, was also noted in BM-derived monocytes/macrophages (Figures 3B,D,F; *N* = 3/group).

### Sex-related differences in neutrophil and platelet involvement in IR injured hearts with neutrophil adhesion reduced in both sexes with IL-36RA

**Figure 4:**
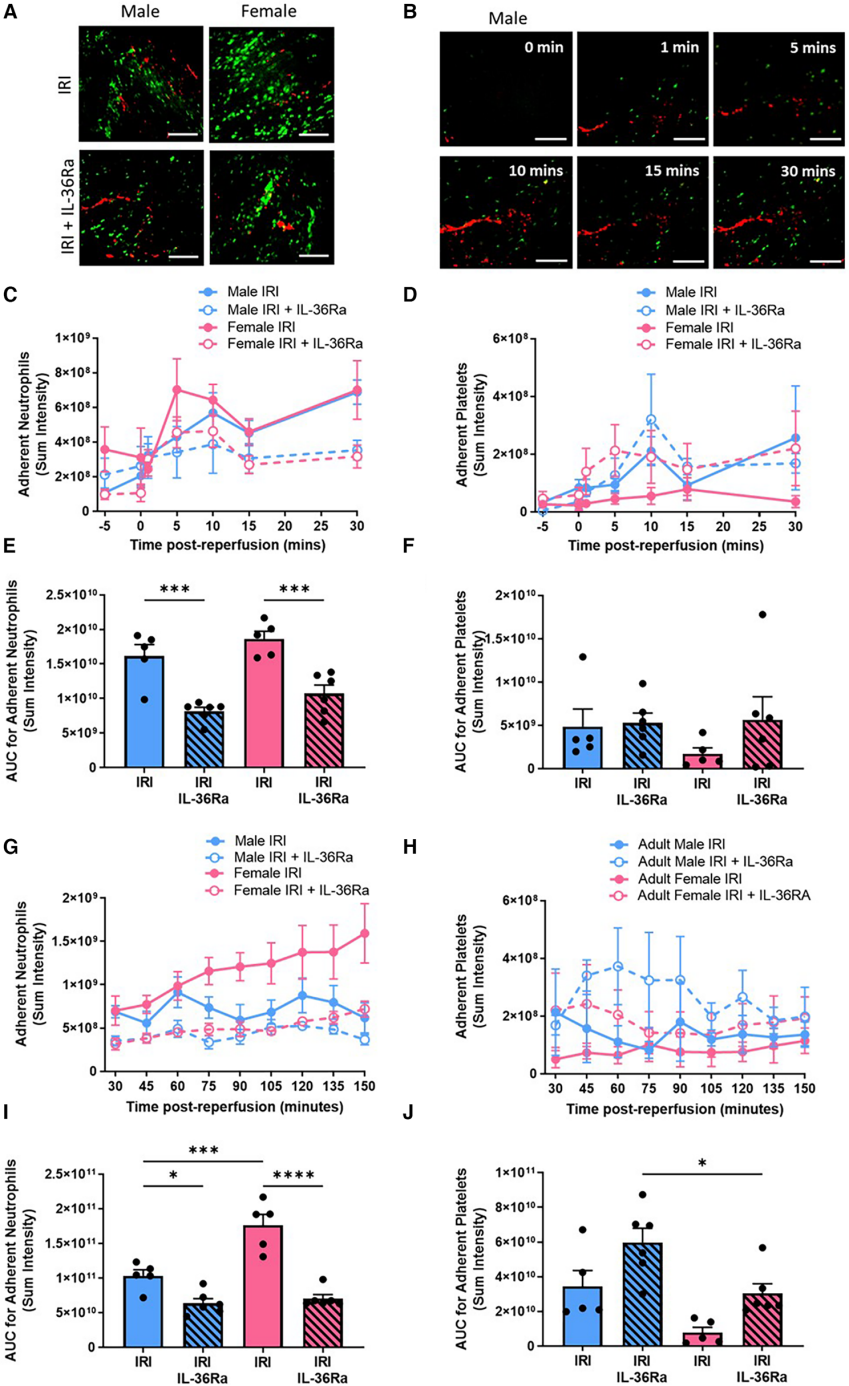
Sex-related differences in neutrophil and platelet involvement in IR injured hearts with neutrophil presence reduced in both sexes by IL-36R inhibition. An IL-36 receptor antagonist (IL-36Ra; 15 μg/mouse) was injected intra-arterially at 10 min pre-reperfusion and at 60 min post-reperfusion in male and female mice. Representative intravital images of the beating heart showing adherent neutrophils (green) and platelets (red) in the coronary microcirculation at (**A**) 120 min post-reperfusion and (**B**) within the first 30 min of reperfusion. (**C–F**) Quantitative analysis of the intravital data and the AUC analysis for adherent neutrophils and platelets over a time course of 30 min post-reperfusion. (**G–J**) Quantitative analysis of the intravital data and the AUC analysis for adherent neutrophils and platelets over a time course of 150 min post-reperfusion. Scale bar = 100 µm. IRI *N* = 5/group; IRI + IL-36Ra *N* = 6/group. *p < 0.05, **p < 0.01, ***p < 0.001, ****p < 0.0001 as determined using a one-way ANOVA followed by a Tukey's post-hoc test. All graphs: male = blue; female = pink.

No significant sex-related differences in neutrophil or platelet presence were observed within the first 30 min post-reperfusion. In both sexes, neutrophil adhesion occurred immediately after reperfusion was initiated and increased to a similar extent during the first 30 min (Figures 4A–F; *N* = 5–6/group). Thereafter, neutrophil recruitment was significantly (*p* < 0.001) higher in female injured hearts compared to males. Furthermore, in male hearts neutrophil presence plateaued after 30 min of reperfusion but continued to increase throughout the 150 min imaging period in females hearts (Figures 4G,I; *N* = 5–6/group). Whilst neutrophil adhesion was primarily confined within coronary capillaries, in some female mice they also appeared as clusters within larger blood vessels. The number of free-flowing neutrophils was significantly decreased (*p* < 0.01—data not presented) in male injured hearts (AUC = 2907.7) when compared to their female counterparts (AUC = 1246.6). In contrast to neutrophil adhesion, the presence of platelet aggregates was higher in male injured hearts, particularly at certain time points post-reperfusion. Microthrombi were not generally observed in the larger vessels but formed primarily within coronary capillaries. In some male hearts, they often occupied significant lengths of affected capillary with numerous smaller and more rounded platelet aggregates dispersed throughout the field of view (Figures 4H,J; *N* = 5–6/group).

In both sexes, IL-36Ra treatment significantly (*p* < 0.001) reduced neutrophil adhesion within the first 30 min of reperfusion and continued to exert this anti-inflammatory effect throughout the entire imaged reperfusion period of 150 min (Figures 4E,I). IL-36Ra made no significant impact on platelet presence within male or female injured hearts within the first 30 min of reperfusion. Thereafter, IL-36Ra treatment did show a general trend to increase platelet microthrombi in both sexes, with significantly (*p* < 0.05) greater numbers noted in treated male hearts when compared to female hearts (Figures 4H,J).

**Figure 5:**
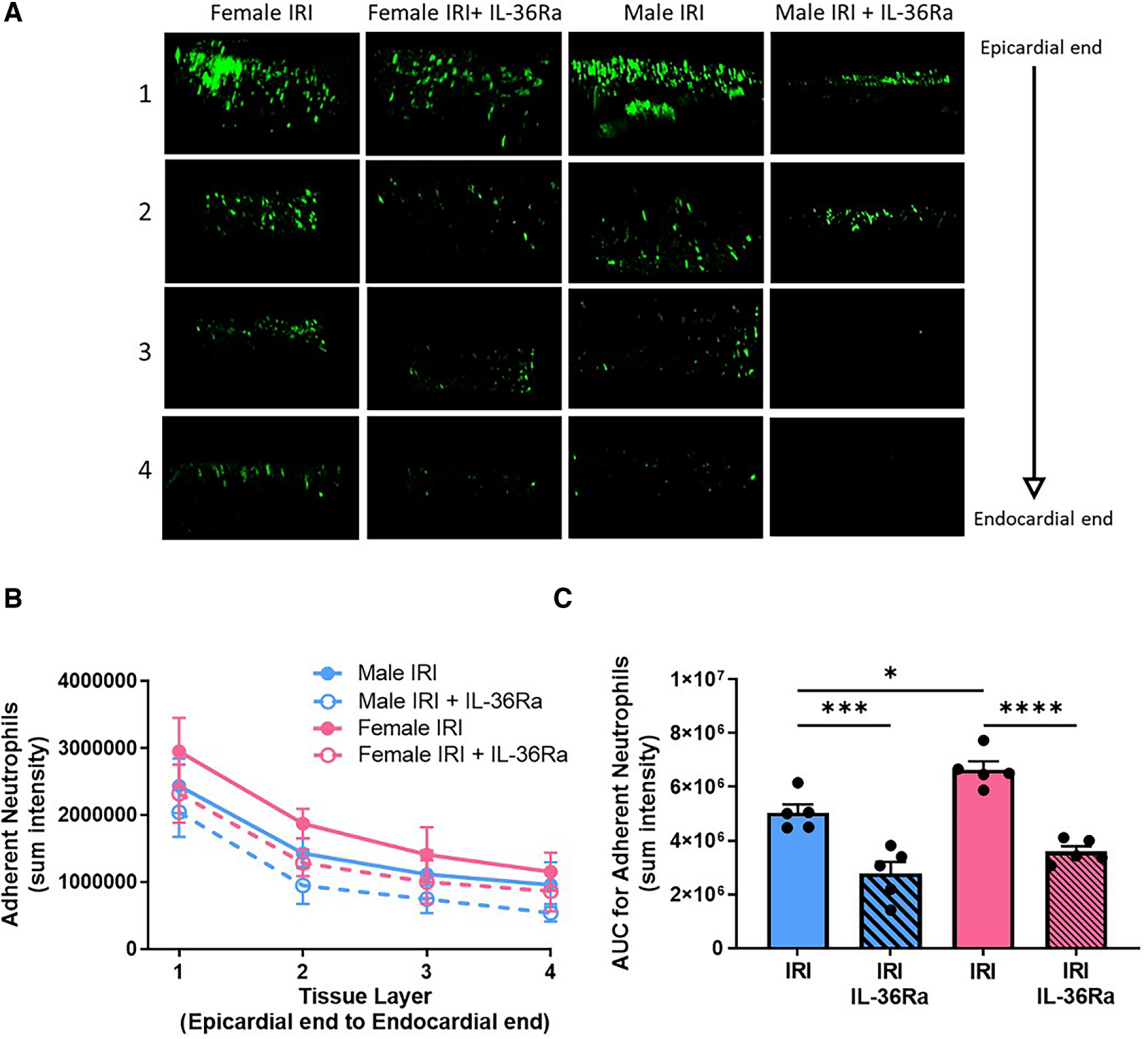
Neutrophil presence reduced throughout the depth of the IR injured left ventricle in both sexes by IL-36R inhibition. An IL-36 receptor antagonist (IL-36Ra; 15 μg/mouse) was injected intra-arterially at 10 min pre-reperfusion and at 60 min post-reperfusion in male and female mice. The left ventricle was vibratome sectioned into four 300 μm sections and imaged using a multiphoton microscope. (**A**) Representative Z-stack multiphoton images of neutrophils (green) in the four sections taken from the outermost layer closest to the epicardium (row 1), outer myocardial layer (row 2), inner myocardial layer (row 3) and innermost layer closest to the endocardium (row 4). (**B,C**) Quantitative analysis of the multiphoton data at various depths and the corresponding AUC analysis for adherent neutrophils. *N* = 5/group. *p < 0.05, ***p < 0.001, ****p < 0.0001 as determined using a one-way ANOVA followed by a Tukey's post-hoc test. All graphs: male = blue; female = pink.

*Ex vivo* multiphoton imaging confirmed the significantly (*p* < 0.05) higher neutrophil recruitment in female injured hearts when compared with male injured hearts and that this was true throughout the thickness of the ventricular wall. However, the highest numbers of neutrophils in response to injury occurred within the outermost 300 μm layer in both sexes. Multiphoton imaging confirmed the ability of IL-36Ra to decrease neutrophil presence in all layers of the ventricular wall in both males (*p* < 0.001) and females (*p* < 0.0001) (Figures 5A–C; *N* = 5/group).

### Functional capillary density and ventricular perfusion improved in both sexes with IL-36RA

**Figure 6:**
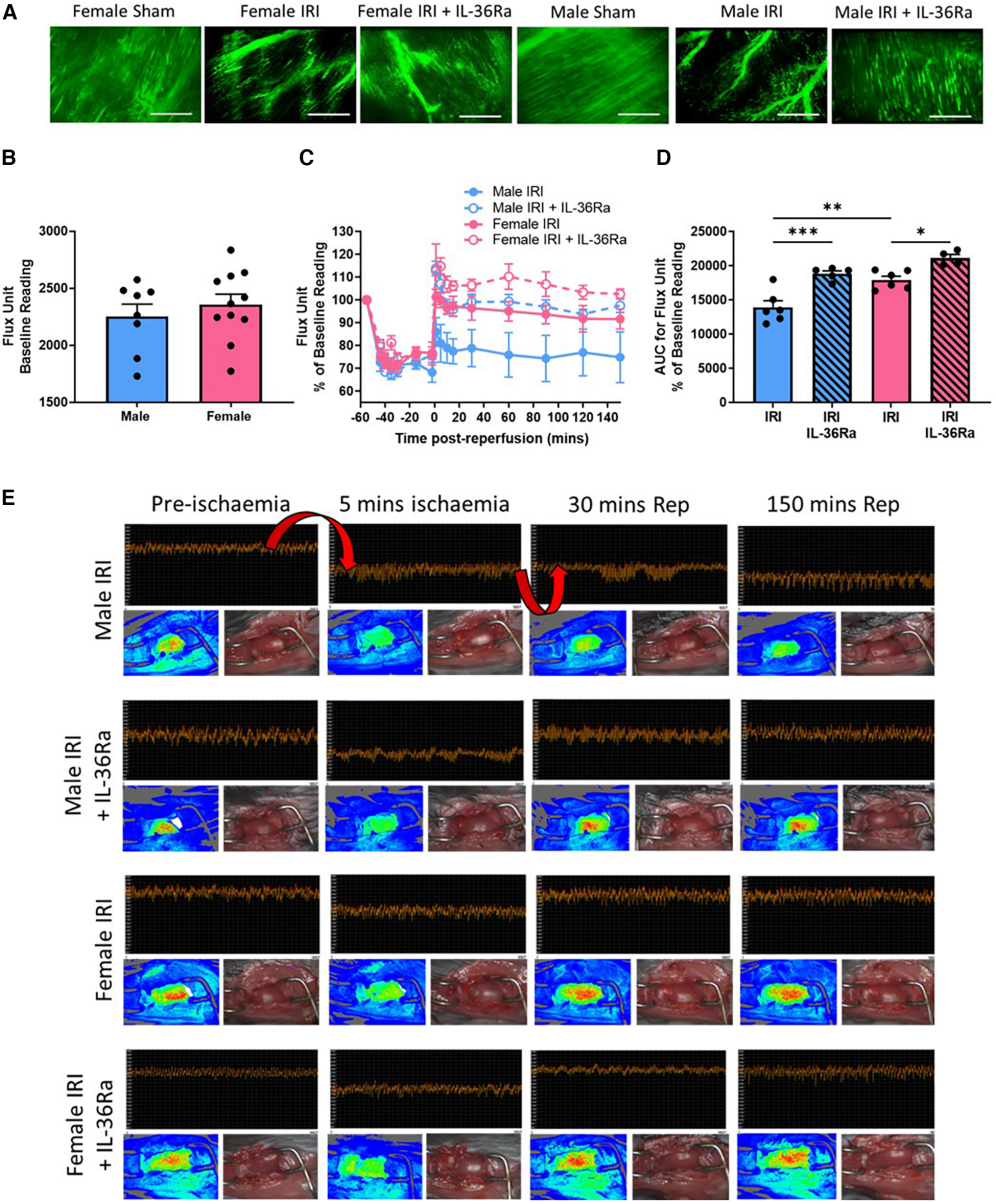
FCD and ventricular perfusion decreased with IR injury in both sexes but was worse in male mice—improved with IL-36R inhibition in both sexes. An IL-36 receptor antagonist (IL-35Ra; 15 μg/mouse) was injected intra-arterially at 10 min pre-reperfusion and at 60 min post-reperfusion in male and female mice. (**A**) Representative intravital images of FITC-BSA perfused coronary microvessels (green) at 150 min in male and female sham hearts or at 150 min post-reperfusion. Patchy black areas represent areas devoid of perfusion. *N* = 3/group. (**B**) Quantitative analysis of baseline diastolic flux (perfusion) unit readings obtained using LSCI prior to injury. Male *N* = 8; Female *N* = 11. (**C,D**) Quantitative time-course analysis of diastolic flux unit readings as a percentage of baseline values obtained by LSCI and the corresponding AUC curve. (**E**) Representative LSCI images showing continuous flux data, flux heat maps and a corresponding photo of the beating heart. Arrows highlight the expected decreased ventricular perfusion during ischaemia and the rise in perfusion (or lack of) during reperfusion. IRI = *N* = 6/group; IRI + IL-36Ra *N* = 4/group. Scale bar = 100 μm. *p < 0.05, **p < 0.01, ***p < 0.001 as determined using a one-way ANOVA followed by a Tukey's post-hoc test. All graphs: male = blue; female = pink.

An extensive network of FITC-BSA perfused capillaries was observed in sham hearts of both sexes paralleling the arrangement of muscle fibres with cross-connections along their length. Focussing up and down on the field of view showed no areas devoid of perfused capillaries. Well perfused medium-sized vessels were also visible in some fields of view. In contrast, in both sexes, IR injury was associated with an increase in the number of patchy areas with no perfusion as evidenced by a lack of FITC-BSA fluorescence. Qualitatively this decreased FITC-BSA appearance, indicative of reduced FCD, appeared worse in male injured hearts. Treatment using IL-36Ra improved FCD in both male and female injured hearts although some areas of devoid of perfusion were still visible (Figure 6A; *N* = 3/group).

Baseline flux readings obtained by LSCI demonstrated no significant difference in overall left ventricular (LV) perfusion between the sexes although it was mildly higher in female hearts (Figure 6B; *N* = 8–11/group). As expected, ischaemia decreased blood flow to the affected LV following LAD artery ligation in both sexes. Although perfusion returned to baseline pre-ischaemic levels upon reperfusion in female hearts, it failed to do so in male hearts. Indeed, blood flow was significantly (*p* < 0.01) lower in male injured hearts when compared to female injured hearts (Figures 6C–E; *N* = 4–6/group). In IL-36Ra treated mice, reperfusion was accompanied by a hyperaemic response, which plateaued but remained above pre-ischaemic baseline values at all-time points in both sexes (Figures 6B–E). Indeed, LV perfusion was significantly enhanced in IL-36Ra treated male (*p* < 0.001) and female (*p* < 0.05) injured hearts when compared to their respective non-treated injured hearts (Figure 6D).

### Infarct size, oxidative damage and VCAM-1 expression decreased in both sexes with IL-36RA

**Figure 7:**
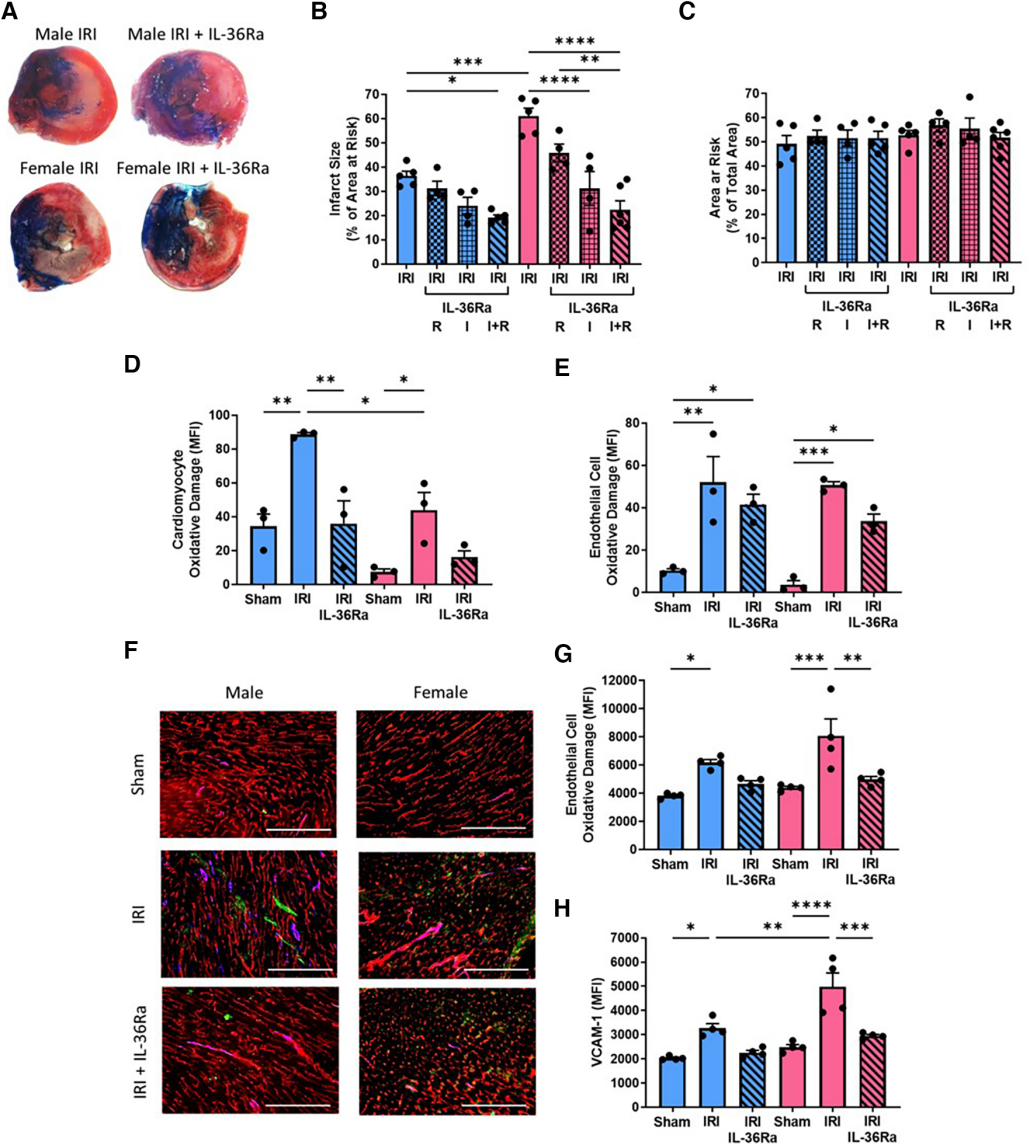
Infarct size, cardiomyocyte/endothelial cell oxidative damage and VCAM-1 expression decreased in both sexes with IL-36Ra. An IL-36 receptor antagonist (IL-36Ra; 15 μg/mouse) was injected intra-arterially either during ischaemia at 10 min pre-reperfusion (*I*), 60 min post-reperfusion (*R*) or at both time points (I + R) in male and female mice. (**A**) Representative images of TTC-stained infarcts. (**B,C**) Quantitative analysis of infarct size and area at risk (AAR). *N* = 4-6/group; IRI + single treatment *N* = 4/group; Male IRI + double treatment *N* = 5; Female IRI + double treatment *N* = 6. (**D,E**) Hearts were collected from male and female sham, IR injured, and IR injured + IL-36Ra treated mice, collagenase digested and analysed flow cytometrically for oxidative damage in CMs and ECs. *N* = 3/group. (**F**) Representative images from these mice immunostained with an anti-CD31 (red), anti-DNA/RNA oxidative damage (green) and anti-VCAM-1 (blue) antibody. (**G,H**) Quantitative analysis of these immunofluorescent images for endothelial oxidative damage and VCAM-1 expression. *N* = 4/group. Scale bar = 200 μm. *p < 0.05, **p < 0.01, ***p < 0.001, *p* < 0.0001 as determined using a one or two-way ANOVA followed by a Tukey's post-hoc test. All graphs: male = blue; female = pink.

Infarct size was significantly (*p* < 0.001) larger in female injured hearts when compared to male injured hearts. This was reduced in male mice receiving single and double doses of IL-36Ra but only attained statistical significance (*p* < 0.05) with the double dose when compared to non-treated injured hearts. Infarct size was also reduced in female mice receiving single and double doses of IL-36Ra, attaining statistical significance (*p* < 0.0001) with the single dose delivered during ischaemia and with the double dose delivered during ischaemia and reperfusion. The area at risk was not significantly different across all groups (Figures 7A–C; *N* = 4–6/group).

Flow cytometric analysis of digested heart cells demonstrated significant CM oxidative damage in both male (*p* < 0.01) and female (*p* < 0.05) injured hearts when compared to sham hearts, but this was significantly (*p* < 0.05) greater in males when compared to females. This was reduced in both sexes with IL-36Ra treatment (Figure 7D; *N* = 3/group). Flow cytometric analysis of digested heart cells demonstrated significant but similar EC oxidative damage in the injured hearts of both sexes, which was also reduced in both with IL-36Ra treatment (Figure 7E; *N* = 3/group). These results were confirmed with immunostaining of heart tissue (Figures 7F,G). Immunostaining also demonstrated significantly increased expression of VCAM-1 in male (*p* < 0.05) and female (*p* < 0.0001) injured hearts, but this was significantly (*p* < 0.01) greater in females when compared to males. This was also reduced in both sexes with IL-36Ra treatment (Figures 7F,H; *N* = 3–4/group).

## Discussion

Sex-related differences in the innate and adaptive immune responses are well recognised. This makes consideration of the effects of sex on the coronary microcirculation critical as it is highly responsive to, and a vital participant in, the inflammatory process. This study provides the following original contributions on sex-related perturbations taking place within the IR injured coronary microcirculation *in vivo*:

• FCD was reduced in both sexes but more so in male hearts
• Males did not restore ventricular perfusion to baseline values post-reperfusion
• Females had a greater burden of inflammation which could be explained by their higher basal and inducible expression of IL-36, IL-36R and VCAM-1. They also had larger infarcts.
• IL-36R antagonism reduced neutrophil presence and infarct size to a similar degree in males and females making it an ideal therapeutic approach for use in both sexes. This was despite the fact this treatment increased platelet presence in male hearts and therefore supports the notion that neutrophils likely play a critical role in infarction.

A number of studies suggest any differences in the outcomes after MI between female and male patients are mainly due to the fact that women are 5–10 years older and thus more comorbid at the time of acute presentation. Indeed, when adjusting for relevant covariables, including age, these studies suggest that no differences in outcomes after the acute event exists (18). However, in contrast to these studies, others have demonstrated that that the female sex is an independent predictor of poorer cardiovascular outcomes after primary PCI (19, 20). Indeed, in-hospital, 30-day and 1- year mortality is higher is females than males. An interesting and detailed study in STEMI patients by Cenko and colleagues showed poorer outcomes and higher mortality in females but this was only true for younger women (<60 years) when compared to younger men, whereas in the older population (60–74 or >75 years) the mortality rate for women and men was similar (21). There are many reasons for this sex-related discrepancy including the possibility that this might be linked to an exaggerated inflammatory response in females. Our study is the first to directly image the beating heart *in vivo* to demonstrate an approximately 2-fold increased neutrophil recruitment in the age-matched female coronary microcirculation after reperfusion injury when compared to males. Moreover, unlike in male mice where neutrophil infiltration plateaued, in female mice this continued to increase throughout the imaging period. Hence, the impact of sex on inflammatory perturbations within the injured coronary microcirculation was not negligible.

### Sex-related differences in neutrophils

Increased neutrophil adhesion in injured female hearts may be linked to our observed greater up-regulation of endothelial VCAM-1 in this sex or to previously published sex-related structural and functional changes in neutrophils themselves (22). Gupta and colleagues performed mRNA-sequencing on human neutrophils and demonstrated significant sex-related differences in gene expression, with 106 genes up-regulated and 128 genes down-regulated in female neutrophils compared to male neutrophils. Specifically, they demonstrated that female neutrophils were enriched in genes related to interferon signalling pathways and were thus more hyperresponsive to interferons than male neutrophils. Female neutrophils also existed in a basally activated state leading to greater pro-inflammatory responses, including greater ability to form neutrophil extracellular traps (NETs). They concluded that this would endow female neutrophils with an increased ability to respond to danger signals and acquire a more pro-inflammatory phenotype with augmented levels of TNF synthesis (23). Similar data was also acquired in mice in which male neutrophils appeared to have more degranulation activity, as evidenced by higher levels of the neutrophil protein elastase, but female neutrophils exhibited greater NETosis (24). These observations, combined with our enhanced neutrophil recruitment in female hearts, may led to greater neutrophil mediated myocardial damage in females in sterile IR injuries. Indeed, infarct size was larger in female mice in the current study.

### Sex-related differences in platelets

In contrast to the neutrophil data, an approximately 3-fold increase in platelet presence was demonstrated in the male injured coronary microcirculation compared to females, with most notable differences noted in the first 30 min of reperfusion. These results do not conform to the general consensus that females have a more hypercoagulable profile (25). Indeed, a recent study by Kim and colleagues showed that during health, human platelets from females were more reactive to the platelet agonists adenosine diphosphate (ADP), thrombin receptor activatory peptide (TRAP-4) and the thromboxane A_2_ mimetic U46619 than platelets from males. However, the same study demonstrated a switch from these observations in health, with platelets isolated from male patients prior to PCI for MI being 1.6-fold more reactive to ADP and 2.7-fold more reactive to TRAP-4 compared to female platelets. They confirmed their human data in platelets from healthy and MI mice (26). These observations may explain our findings of increased microthrombus presence within the coronary microvessels of male IR injured mice.

### Sex-related differences in perfusion

Intravitally, we noted that in coronary microvessels occupied by platelet aggregates, no free-flowing neutrophils could be observed passing through them, suggesting these microthrombotic events may have been occlusive. This may explain poor FITC-BSA perfusion and overall ventricular perfusion in male hearts. In contrast, in female injured hearts, despite significant neutrophil presence, ventricular blood flow returned to baseline levels upon reperfusion. As far as we are aware, there have been no studies that have experimentally investigated, using either intravital or LSCI imaging, sex-related myocardial perfusion disturbances post-reperfusion injury and so our findings provide new information about dynamic microcirculatory perturbations beyond thromboinflammatory differences between the sexes. Our experimental findings are supported by a recent clinical study by Nickander and colleagues who used cardiovascular magnetic resonance imaging to identify lower basal myocardial perfusion, as well as significant decreases following adenosine-induced stress, in male participants (27).

It is possible that differences in thrombotic and inflammatory events could be due to sex-related differences in CM, and particularly EC, oxidative damage. However, we only noted increased CM oxidative damage in male injured hearts when compared to females, with similar EC damage between the two sexes. Several studies have shown an enhanced oxidative stress response specifically in males. For example, Barp and colleagues showed increased myocardial oxidative stress, as assessed by lipid peroxidation, and decreased anti-oxidants such as superoxide dismutase, in male vs. females rats and suggested this to be related to the anti-oxidant role of oestrogen and not testosterone in the heart (28). Similarly, evidence of higher basal levels of *in vivo* markers of oxidative stress in males compared to females has also been noted in human studies (29). Vascular oxidative stress is well known to be associated with elevated thrombosis due to suppressed bioavailability of anti-thrombotic nitric oxide (30). Since we did not note sex-related differences in EC oxidative stress, it is not clear why male hearts had a greater propensity to develop microthrombi and so this would need further investigation.

### Sex-related differences in IL-36/IL-36R expression

IL-36 is typically one of the most upstream and up-regulated pro-inflammatory cytokines released upon cellular necrosis (31). This study shows that basal IL-36R and IL-36α/β expression was low in both sexes, consistent with previous observations (32) and that whilst their expression increased in both sexes following injury, this was more pronounced in female hearts. Since human heart tissue would not have been possible to obtain from MI patients, for proof-of-principle, we investigated IL-36R/IL-36 in male and female tissue from patients undergoing LVAD implantation. Again, similar sex-related differences were noted with greater presence in female hearts. As far as we are aware, this is the first demonstration that members of this novel cytokine pathway are expressed differentially in both mouse and human male and female hearts. Although sex-related cytokine receptor changes have not been studied extensively, an enhanced production of inflammatory cytokines and how their synthesis and release varies between sexes during different diseases has been demonstrated. Some of these studies show increased release in males, but most confirm our results of increased production in females (33). A common finding in diseases where IL-36 cytokines contribute to pathology is the remarkable presence of neutrophils. We have previously demonstrated intravitally that IL-36 agonists topically applied to the heart were potently pro-inflammatory, something not previously shown *in vivo* in any organ let alone the heart (14). Hence, our demonstrated increased expression of pro-inflammatory IL-36/IL-36R in injured female hearts may explain the increased levels of neutrophils within the coronary microcirculation of female rather than male hearts.

### Vasculoprotective efficacy of IL-36RA in both sexes

To date, several clinical trials have been conducted which target inflammatory cytokines post-MI including IL-1, IL-6, and TNFα (11, 34–38). Since no studies had evaluated the therapeutic effectiveness of targeting IL-36, we sought to identify if systemic administration of IL-36Ra was protective against the deleterious effects of IR injury in male and female mice. This was considered important since studies have shown sex-related differences in the efficacy of drugs with anti-inflammatory properties (39). Our novel data showed that administration of IL-36Ra was associated with an approximately 30% and 50% reduction in neutrophil recruitment in both male and female injured hearts respectively. Interestingly, the number of adherent neutrophils at the end of the imaged reperfusion period was similar in both sexes suggesting IL-36Ra, at the dose used, could only inhibit neutrophil adhesion to a certain level. The anti-inflammatory ability of IL-36Ra is increasingly recognised and recently demonstrated in psoriasis and rheumatoid arthritis, with neutrophil reduction also noted in intestinal disease in IL-36R knock-out mice (40).

We and others have shown myocardial reperfusion to be associated with a worsening of coronary flow to the heart (41). In the current study, LSCI showed this to be worse in male injured mice compared to female injured mice. However, IL-36Ra treatment not only improved blood flow to the reperfused LV, but actually enhanced it above baseline values in both male and female mice. This transient initial hyperaemic response was accompanied by a more sustained increase in perfusion than in non-treated mice, highlighting the importance and benefits of timely anti-neutrophil interventions. Our data supports the observations of Kerckhoven and colleagues, who also showed that use of the anti-inflammatory methylprednisolone could improve FCD following myocardial IR injury in rats (42). However, this was quantitated by staining ECs with lectin in paraffin sections in recovery mice at 21 days MI. In contrast, LSCI provides a more functionally relevant, continuous, and dynamic readout of blood flow in the beating heart *in vivo*.

Our data provides novel mechanistic insights into how inhibition of IL-36/IL-36R signalling attenuated oxidative stress and VCAM-1 expression in both sexes which led to decreased neutrophil recruitment in the coronary microcirculation. This impacted tissue infarction reducing it to similar levels in both sexes despite increased platelet presence in male hearts and increased neutrophil presence in female hearts. Our data support recent observations of reduced oxidative stress in IL-36R knockout rats undergoing cardiopulmonary bypass (43). Importantly we show that similar pathophysiological mechanisms are targeted and down-regulated by IL-36Ra in both sexes post-IR injury. Since infarct size is strongly associated with prognosis (44), the ability of IL-36Ra to reduce this to equal levels in both sexes suggests targeting the IL-36 pathway may be beneficial to both sexes. Although this benefit is likely linked to the anti-neutrophil effect of IL-36R antagonism, it is unclear whether there are other additional cardioprotective effects of IL-36Ra. However, what was clear from our findings was that the effectiveness of this novel cardioprotective therapy was dependent on its administration prior to reperfusion and enhanced if repeated post-reperfusion. Indeed, inflammatory responses in the coronary microcirculation occurred within minutes of untying the LAD artery ligature but could be targeted by the IL-36Ra if administered during the ischaemic period. This opens the possibility of timely targeting of IL-36 during PCI procedures to be therapeutically efficacious in a clinical setting (45).

## Concluding remarks

There remains an unmet need to discover and optimise novel therapeutic strategies that are effective in improving the current poor prognosis after MI. This is particularly true for female patients where outcomes may be worse for women compared with men. We and others have recommended specific protection of the delicate coronary microcirculation from IR injury. This study is the first to explore and identify notable sex-related differences in the response of the coronary microcirculation to myocardial IR injury *in vivo* Whilst contemporary treatment for MI focuses on anti-platelet strategies, the heightened presence of neutrophils in the reperfused female coronary microcirculation necessitates a more anti-inflammatory approach in women. The well known CANTOS and LoDoCo2 trials have already paved the way for the use of anti-inflammatories, namely IL-1β and colchicine respectively, for the treatment of MI. These trials provided evidence that directly targetting inflammation is beneficial for the secondary prevention of major adverse cardiovascular events. However, our novel data underpins the design of a clinical trial using an IL-36R inhibitor with the primary aim of protecting the coronary microcirculation in the immediate aftermath of PCI-induced reperfusion injury. We also emphasise the importance of early intervention with an IL-36R inhibitor in order to maximise therapeutic effectiveness.

## Data Availability

The original contributions presented in the study are included in the article/Supplementary Material, further inquiries can be directed to the corresponding author.
